# Effect of educational training about CAM-ICU on nurses’ knowledge, confidence, and practice to detect delirium among critically ill patients in intensive care unit

**DOI:** 10.1186/s12912-025-03395-0

**Published:** 2025-06-27

**Authors:** Mona Ibrahim Hebeshy, Samia Gaballah, Eman Ragab Elsayed

**Affiliations:** Medical-Surgical Nursing, Faculty of Nursing, Suez Canal University, 4.5 KM the Ring Road, Ismailia, 41522 Egypt

**Keywords:** ICU delirium, CAM-ICU, Nursing education, Self-confidence, Knowledge, Delirium management, Documentation accuracy

## Abstract

**Background:**

Delirium in the intensive care unit (ICU) is a serious complication associated with increased mortality, prolonged hospital stays, and long-term cognitive decline. Early detection through standardized assessment tools is critical to improving patient outcomes. This study evaluated the effect of an educational training program on ICU nurses’ self-reported knowledge, perceived self-confidence, and comfort levels in using the Confusion Assessment Method for the ICU (CAM-ICU), as well as their observed performance in delirium assessment and documentation.

**Methods:**

A quasi-experimental single-group pretest-posttest design was conducted with 50 ICU nurses in an Egyptian ICU. Participants received a four-week educational program on delirium and CAM-ICU use. Knowledge, confidence, and comfort levels were measured before and after training using validated questionnaires. Post-training performance was assessed using an observational checklist evaluating assessment accuracy, documentation completeness, and consistency. Paired t-test was used for data analysis.

**Results:**

ICU nurses’ knowledge and confidence in using the CAM-ICU significantly improved after training (*p* < .001). Post-training, assessment accuracy (95.5%) and documentation completeness (91.3%) were high, while practice consistency (79%) met the satisfactory threshold (≥ 75%), indicating satisfactory performance in delirium assessment and documentation using CAM-ICU.

**Conclusions:**

The educational training significantly improved ICU nurses’ knowledge, perceived self-confidence, comfort levels, and practical skills in delirium assessment using the CAM-ICU, leading to more accurate and consistent screening practices. Structured, interactive training should be integrated into ICU education to promote standardized delirium assessment in ICU settings.

**Implications for practice:**

Integrating structured delirium education into ICU training can promote adherence to best practices, ensuring timely detection and intervention. Future research should examine long-term skill retention and its effect on patient outcomes.

**Clinical trial number:**

Not applicable.

## Background

Delirium is one of the most frequent syndromes in intensive care units (ICUs). It is characterized by a rapidly developing disturbance in consciousness and cognition, with symptoms that fluctuate throughout the day [1]. Delirium can present as hypoactive, hyperactive, or mixed subtypes [1]. Hyperactive delirium, the most frequently observed subtype, is characterized by psychomotor agitation, restlessness, and sometimes aggressive behavior, often leading to misdiagnosis as a psychotic disorder or agitated dementia. In contrast, hypoactive delirium presents with reduced psychomotor activity, apathy, and slowed speech, giving the appearance of sedation. This subtype is frequently underrecognized, resulting in delayed treatment and worse patient outcomes [2–5].

Given its varied presentations, delirium often goes undiagnosed, contributing to severe clinical consequences. Delirium can lead to adverse outcomes, including prolonged mechanical ventilation, increased ICU stays, long-term cognitive impairments, higher costs, and increased mortality. It also raises the risk of hospital-acquired complications, such as pressure ulcers and persistent cognitive deficits, as well as a higher likelihood of discharge to long-term care facilities [6–8].

The incidence of delirium in ICUs ranges between 45% and 87%, with a higher prevalence among mechanically ventilated patients [9–11]. Despite its prevalence, delirium remains under-detected, particularly in resource-constrained settings. Studies indicate that up to 72% of ICU patients go unrecognized when routine screening tools are not used [12]. Notably, 30–40% of delirium cases could be prevented with early intervention [1], highlighting the critical need for consistent assessment.

The Confusion Assessment Method for the ICU (CAM-ICU) has emerged as a widely recognized and validated tool among the various delirium assessment tools. The CAM-ICU evaluates four key features: acute onset or fluctuating course, inattention, disorganized thinking, and altered level of consciousness [13]. With high sensitivity (93–100%) and specificity (98–100%), it is a reliable tool for ICU nurses, even those without psychiatric training [14–17]. Unlike more complex assessments, the CAM-ICU can be completed in 2–5 min, making it feasible for high-demand ICU environments [18].

The Society of Critical Care Medicine (SCCM) and the American Association of Critical-Care Nurses (AACN) strongly recommend routine delirium monitoring using validated tools like the CAM-ICU [11]. Nurses can use these tools at least once every 8 to 12 h during their shifts to improve early detection and intervention [18]. However, global adherence remains inconsistent due to barriers such as inadequate training, low nurse confidence, and competing clinical priorities [7, 19–21]. These challenges are exacerbated in settings like Egypt, where high nurse-to-patient ratios and limited access to training further hinder effective delirium screening.

Structured educational programs have been shown to address these gaps by enhancing healthcare providers’ knowledge and skills in delirium recognition and management. These programs typically employ multimodal teaching strategies, including interactive lectures, case discussions, hands-on simulation training, and e-learning modules [22–23]. Research by Flagg et al. (2010), Marino et al. (2015), and Ramoo et al. (2018) has demonstrated that such educational interventions significantly improve ICU nurses’ ability to recognize, apply assessment tools effectively, and implement evidence-based interventions, leading to better patient outcomes [24–26].

However, in Egypt, where delirium protocols are not yet standardized, tailored training programs are urgently needed to address this practice gap. Therefore, this study aimed to evaluate the effect of an educational program on ICU nurses’ knowledge and perceived self-confidence in using the CAM-ICU tool. The program was adapted to the Egyptian context, with materials translated into Arabic and aligned with local workflows to maximize feasibility and implementation.

## Aim of the study

This study aimed to evaluate the effect of an educational program on ICU nurses’ knowledge and confidence, and comfort levels in using the CAM-ICU tool.

### Objectives

- To assess ICU nurses’ knowledge and perceived self-confidence and comfort levels in using the CAM-ICU tool before implementing the educational training.
- To measure ICU nurses’ knowledge and perceived self-confidence and comfort levels in using the CAM-ICU tool after implementing the educational training.
- To evaluate the accuracy, completeness, and consistency of ICU nurses’ assessment and documentation practices while using the CAM-ICU after implementing the educational training program.

### Research hypotheses

- **H**_**1**_: There is a significant difference in ICU nurses’ knowledge and perceived self-confidence and comfort levels in using the CAM-ICU tool before and after the educational training program.
- **H**_**2**_: ICU nurses demonstrate satisfactory assessment and documentation practices using the CAM-ICU after the educational training program.

## Methodology

### Study design

This study employed a quasi-experimental single-group pretest-posttest design. A study questionnaire was administered before and after the intervention to evaluate perceived self-confidence and comfort level with ICU delirium care and knowledge. The aim was to determine the effectiveness of a structured training program incorporating the CAM-ICU tool in enhancing nurses’ ability to identify and manage delirium in the intensive care unit.

### Study setting

The study was conducted in the adult medical ICU of Suez Canal University Hospital in Ismailia. This ICU has 16 beds and admits critically ill adult patients with a variety of medical and surgical conditions. The unit is staffed by 86 full-time registered nurses working in three rotation-based shifts per day, maintaining a nurse-to-patient ratio of 1:1 or 1:2.

### Participants

A convenience, nonprobability sample was recruited for this study. The sampling process involved disseminating study information through flyers, staff meetings, and internal communication channels such as hospital bulletin boards. Interested nurses were invited to contact the research team for more details. Eligible participants included registered nurses responsible for evaluating and documenting ICU patient assessments, with more than six months of ICU nursing experience. Nurses were excluded if they had participated in a similar study or attended specialized courses on delirium prevention. Eligible participants provided written informed consent before enrollment.

### Sample size Estimation

The sample size was determined using a priori power analysis for a paired sample t-test (two-tailed) in G.Power version 3.1.9.7 (Faul et al., 2007) [27]. Based on Cohen’s (1988) guidelines, with a medium effect size (d = 0.5), a significance level (α) of 0.05, and a power (1 - β) of 0.95, the minimum required sample size was calculated to be 44 participants. To account for a potential 10% attrition rate, the sample size was increased to 50. The medium effect size of d = 0.5 was chosen based on the expectation of a moderate improvement in nurses’ knowledge and confidence levels post-intervention.

## Measures

This study utilized three tools to measure knowledge of delirium, perceived self-confidence and comfort levels, and an observational checklist of the accuracy of CAM-ICU assessment and documentation practices.

### Delirium knowledge assessment questionnaire

To evaluate the effectiveness of the educational training, we employed a modified self-report questionnaire adapted from validated instruments [14, 17, 28, 29]. The tool comprised two sections: (1) a demographic component including age, gender, educational level, years of clinical experience, and years of experience working in ICU; and (2) a 15-item multiple-choice knowledge assessment evaluating assessing delirium knowledge and assessment techniques, scored dichotomously (1 point per correct response; maximum = 15). The instrument underwent rigorous translation via back-translation methodology with bilingual experts to ensure conceptual and linguistic equivalence, followed by pilot testing (*n* = 5) to verify item clarity. Content validity was assessed by a panel of three experts in critical care nursing, who evaluated the relevance and accuracy of the items. Internal consistency was assessed using Cronbach’s alpha, which yielded a value of 0.88, demonstrating good reliability.

### Perceived self-confidence and comfort levels with providing ICU delirium care scale

A five-item self-reported scale adapted from Marino et al. (2015) was used to measure nurses’ perceived self-confidence and comfort levels in providing ICU delirium care. Nurses rated their agreement with each statement on a scale from 1 “strongly disagree” to 5 “strongly agree” [25]. The scale was translated into Arabic using the back-translation method to ensure linguistic and conceptual accuracy. The translation process involved two independent bilingual translators: one translated the scale from English to Arabic, and the second translated it back to English. Discrepancies were resolved through discussion. The Arabic version was then pilot tested with a small group of ICU nurses (*n* = 5) to assess clarity and relevance. Cronbach’s alpha was calculated to evaluate internal consistency, with a value of 0.85, indicating good reliability.

The scale was administered concurrently with the knowledge questionnaire during the pre-test and post-test phases to assess changes in perceived self-confidence and comfort levels following the educational training.

### Assessment and Documentation practices checklist

To objectively evaluate nurses’ clinical competency in delirium assessment, we developed a standardized observational checklist through a systematic process, beginning with a comprehensive literature review and input from experts in critical care nursing and delirium management. The checklist assessed three key domains: (1) accuracy of CAM-ICU application (4 points), evaluating correct identification of all diagnostic features (acute onset/fluctuating course, inattention, disorganized thinking, and altered consciousness); (2) completeness of delirium documentation (4 points), verifying inclusion of all required items in CAM-ICU worksheets; and (3) consistency of practice (2 points), examining adherence to assessment frequency and time of documentation. A scoring system was employed, with satisfactory performance defined as achieving a score ≥ 75% (7.5/10 points) [30]. The checklist underwent content validation by a panel of experts and was pilot tested with a small group of ICU nurses to ensure clarity and practicality. Interrater reliability was assessed using Cohen’s kappa (κ), yielding a value of 0.85, indicating strong agreement between raters. The finalized checklist was used by trained researchers to evaluate nurses’ CAM-ICU assessments and documentation practices during the study implementation phase.

## Data collection procedure

Data was collected in three phases: pre-test (two weeks before the educational training), implementation (four weeks), and post-test (two weeks after the educational training). The study phases were finished in nine months, starting in September 2023 and ending in May 2024.

During the pre-test phase, informed consent was obtained from eligible participants. They then completed the Delirium Knowledge Assessment Questionnaire and Perceived Self-Confidence, and Comfort Levels with Providing ICU Delirium Care Scale. This phase established baseline levels of knowledge and confidence prior to the educational training.

The implementation phase combined training with clinical integration. Nurses participated in structured educational sessions while simultaneously applying the CAM-ICU in practice. Arabic versions of assessment worksheets were placed at each bedside, with nurses instructed to perform delirium screenings every 12 h for routine cases or every 4 h for sedated or CAM-positive patients. Two trained researchers evaluated clinical implementation through direct observations and documentation reviews using standardized observational checklists. These assessments focused on three key domains: accuracy of CAM-ICU application, completeness of documentation, and consistency with protocols.

**Fig. 1:**
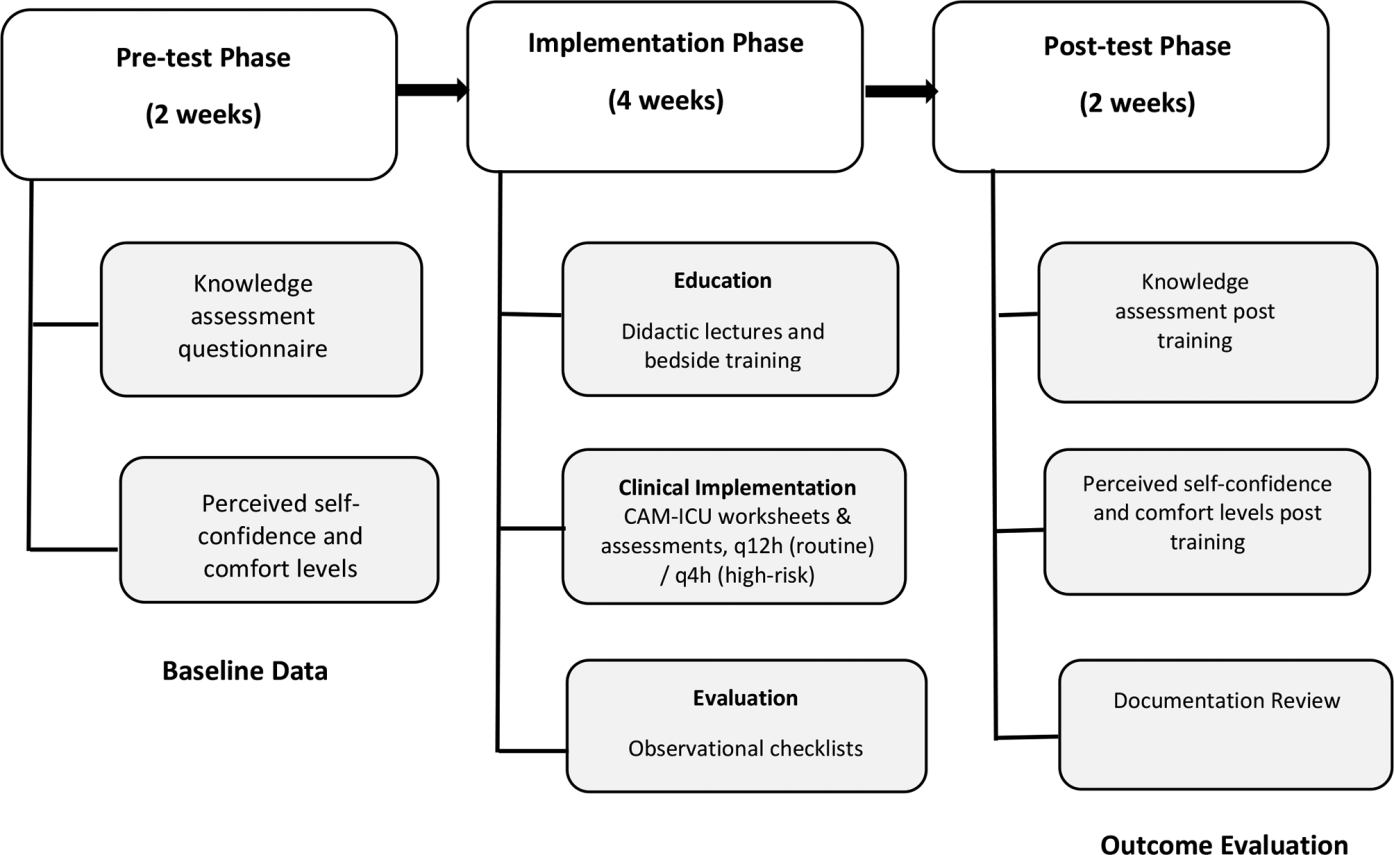
Study design flowchart illustrating the three-phase data collection process: (1) Pre-test Phase (baseline knowledge, self-confidence, and comfort levels assessment), (2) Implementation Phase (education and clinical training with CAM-ICU tool), and (3) Post-test Phase (outcome evaluation and documentation review)

In the post-test phase, nurses completed the same confidence and knowledge surveys administered during the pre-test phase. Researchers collected the CAM-ICU worksheets and flow sheets completed during the implementation phase. These documents were securely stored in a locked location to ensure participant privacy. No patient or nurse identifying information was included in the collected data. Pre and post-test data were compared to assess changes in nurses’ knowledge, confidence, and documentation practices. Flowchart of the pre-test–intervention–post-test study design, outlining the three-phase process (See Fig. 1).

### Educational training program

The educational training program was designed based on a comprehensive literature review of current evidence on ICU delirium management and input from experts in delirium care. The program aimed to enhance nurses’ knowledge, skills, and confidence in assessing and detecting ICU delirium using the CAM-ICU tool. The specific objectives of the program were as follows:

1. To improve nurses’ understanding of ICU delirium, including its definition, pathophysiology, risk factors, and clinical significance.
2. To enhance nurses’ ability to recognize and assess delirium using the CAM-ICU tool.
3. To emphasize the importance of early detection and management of delirium in improving patient outcomes.
4. To provide hands-on training in applying the CAM-ICU tool and documenting findings accurately.

The program was delivered over a four-week period and included both theoretical and practical sessions. The theoretical sessions consisted of two hours of interactive lectures covering the definition, pathophysiology, risk factors, and challenges of ICU delirium, as well as step-by-step guidance on using the CAM-ICU tool. Practical training involved two hours of hands-on bedside sessions, where nurses applied the CAM-ICU tool with actual patients under the supervision of trained researchers. Unlike many existing studies that rely on simulations or manikins, this study included real patient interactions during training, providing a more authentic and impactful learning experience. Video demonstrations (6–7 min each) were also used to illustrate real-world assessment techniques and reinforce key concepts. To ensure accessibility and continued learning, training materials, including PowerPoint slides, CAM-ICU worksheets, and video resources, were shared with participants via WhatsApp following the completion of the in-person sessions. The training materials, including the CAM-ICU tool, were translated into Arabic and adapted to align with regional clinical practices, ensuring greater relevance and applicability for the participating nurses. This cultural and linguistic adaptation was critical to addressing the unique needs of the local healthcare context and maximizing the program’s effectiveness.

While several tools are available for delirium detection, the CAM-ICU was chosen for its widespread use, high accuracy in detecting ICU delirium, and practicality in busy clinical settings [31]. Its structured approach and ease of use made it particularly suitable for educational interventions, enabling nurses to confidently integrate delirium assessments into their daily practice.

### Ethical consideration

This study was conducted in accordance with the ethical standards of the institutional research committee and with the 1964 Declaration of Helsinki. Ethical approval for the study was obtained from the Faculty of Nursing Research Ethics Committee and the hospital administrators in the study settings. Written informed consent was secured from all participants before data collection. The study’s purpose, procedures, and confidentiality measures were explained in detail to participants. Participation was entirely voluntary, and participants were informed of their right to withdraw from the study at any time without consequences. All data were anonymized and securely stored. Only authorized researchers had access to the data, which would be retained for five years before being securely destroyed.

### Data analysis

Data was examined for error and completeness. Each item and option was coded using numbers or unique identifiers before entering spreadsheets in Excel. All the coded items were entered on the spreadsheets and then exported to SPSS (version 29). Each variable was critically examined for outliers by reviewing the data and computing descriptive statistics. Normality was confirmed using Shapiro-Wilk tests (all variables W > 0.95, *p* > .05), supporting parametric test assumptions. Descriptive statistics were used to characterize the study sample, including demographic information (age, gender, years of experience, and educational level). Frequencies and percentages were calculated for categorical variables (e.g., gender, educational level). Means and standard deviations were calculated for continuous variables (e.g., age, years of experience, knowledge scores, and confidence scores). The Paired T-test was used to assess the effect of educational training on nurses’ knowledge and confidence. Missing data were handled using listwise deletion for incomplete surveys. Statistical significance was set at *p* < .05.

## Results

### Demographics of participants

Table 1 presents the demographic characteristics of the 50 ICU nurses who participated in the study. The majority were aged 25–34 years (64%) and female (70%). Most participants held a Bachelor of Science in Nursing (BSN) degree (62%) and had 1–5 years of general nursing experience (56%). Regarding ICU-specific experience, 62% had 1–4 years, 32% had 5–9 years, and 6% had more than 10 years of ICU practice.

**Table 1:** Demographic characteristics of participants (*n* = 50)

Descriptive Characteristics		*n* (%)
Age	18–24	12 (24)
	25–34	32 (64)
	35–44	6(12)
Gender	Male	15 (30)
	Female	35 (70)
Level of education	Diploma	19 (38)
	BSN	31(62)
Years of experience in nursing	< 1 1–5 6–10 11–15 > 15	2 (4) 28 (56) 9 (18) 5 (6) 10 (12)
Years of ICU experience	1–4 > 4–10 > 10	31 (62) 16 (32) 3 (6)

## Effect of educational training on knowledge and perceived self-confidence and comfort levels

Paired sample t-tests were conducted to assess the effect of the CAM-ICU educational program on nurses’ knowledge and perceived self-confidence in delirium assessment. As shown in Table 2, the educational training program significantly improved ICU nurses’ knowledge and confidence in using the CAM-ICU. The mean knowledge score increased from 4.64 (SD = 1.80) pre-training to 9.58 (SD = 2.06) post-training, The difference was statistically significant, *t*(49) = 13.53, *p* < .001, 95% CI [4.21, 5.67] demonstrating a large effect (*p* < .001, Cohen’s *d* = 1.44). Similarly, nurses’ self-confidence and comfort levels with delirium care increased significantly, with the mean score rising from 13.18 (SD = 1.77) pre-training to 20.34 (SD = 1.39) post-training *t*(49) = 20.39, 95% CI [6.47, 7.84], (*p* < .001, Cohen’s *d* = 2.31). These results indicate that the training program effectively enhanced both theoretical knowledge and practical confidence in using the CAM-ICU tool.

**Table 2:** Paired T-test comparing pre-test and post-test scores on knowledge and perceived self-confidence and comfort levels (*n* = 50)

Variable	Pre-test Mean (SD)	Post-test Mean (SD)	Mean Difference (SD)	SEM	95% CI of Difference		t-value	*p*-value
					Lower	Upper		
Knowledge	4.64 (1.80)	9.58 (2.06)	-4.94 (2.58)	0.25	-5.67	4.21	-13.52	< 0.001**
Self-confidence and comfort levels	13.18 (1.77)	20.34 (1.39)	-7.16 (2.41)	0.342	-7.84	6.47	-20.39	< 0.001**

## Post-training CAM-ICU assessment and Documentation performance

In terms of practical application, nurses’ performance in delirium assessment and documentation were evaluated post-training. As shown in Table 3, the results indicated high levels of accuracy in CAM-ICU application (95.5%) and completeness of documentation (91.3%). Although practice consistency was slightly lower (79%), it still met the predefined threshold for satisfactory performance (≥ 75%). These findings suggest that the training program led to strong adherence to clinical protocols for delirium assessment, though some areas, particularly in maintaining consistent practices, may benefit from further reinforcement in future training sessions.

**Table 3:** CAM-ICU assessment and Documentation performance (*N* = 50)

Domain	Mean Score (SD)	Percentage Score	Satisfactory Performance (≥ 95%)
• Accuracy of Application	3.82 (0.38)	95.5%	96% (*n* = 48)
• Documentation Completeness	3.65 (0.49)	91.3%	96% (*n* = 48)
• Practice Consistency	1.58 (0.49)	79%	95% (*n* = 48)
**Total Score**	**9.05 (0.92)**	**90.5%**	**96% (n = 48)**

## Discussion

### Delirium knowledge

This study evaluated the effectiveness of educational training in improving ICU nurses’ knowledge, perceived self-confidence, and practical application of the CAM-ICU tool in delirium assessment. The findings revealed statistically significant improvements across all measured outcomes, reflecting both cognitive and behavioral gains following the educational training.

The significant increase in knowledge scores confirms that structured, context-specific education enhances clinical knowledge and understanding. This finding aligns with adult learning theory, which posits that adults learn most effectively when education is problem-centered, immediately relevant to their work, and grounded in prior experience [32, 33]. The CAM-ICU training was specifically tailored to the ICU context, enabling participants to connect new knowledge with existing clinical practice, thereby enhancing retention and comprehension.

These results are consistent with prior studies demonstrating the positive impact of focused educational interventions on delirium-related knowledge [20, 34, 35, 36]. In Egypt, where delirium education is not routinely embedded in nursing curricula or hospital training programs, such interventions play a critical role in bridging knowledge gaps. Furthermore, constructivist learning theory suggests that knowledge is best acquired when learners actively integrate new information into existing cognitive frameworks, a process supported by the interactive nature of our educational training [37, 38].

#### Perceived self-confidence and comfort levels

Educational training also led to a significant increase in nurses’ self-confidence when assessing delirium. According to Bandura’s self-efficacy theory, confidence increases when individuals develop mastery through repeated practice and success [39]. The hands-on design of the training, including direct application with real patients, provided nurses with experiential learning opportunities that fostered a stronger sense of competence and confidence.

In the Egyptian ICU context, often characterized by high patient loads, staffing shortages, and limited access to continuing professional development, nurses may face barriers that challenge their confidence in handling complex clinical issues like delirium. Providing focused training in such environments not only increases clinical skills but also promotes professional empowerment, which is essential for sustained behavior change and improved patient outcomes.

These findings aligned with previous research showed that structured educational programs can significantly enhance nurses’ confidence in delirium care [40–42]. For example, hands-on CAM-ICU training has been associated with increased comfort levels in identifying at-risk patients [43]. Additionally, systematic reviews of digital and interactive education have demonstrated improvements in delirium-related competencies among healthcare providers and students [44, 45], with long-term benefits in knowledge, behavior, confidence, and patient care outcomes [46].

#### CAM-ICU assessment and Documentation

Post-educational training, nurses demonstrated high accuracy and completeness in CAM-ICU assessments and documentation. However, some inconsistencies in practice may stem from operational barriers commonly found in Egyptian ICUs, such as time constraints, limited supervision, and variability in team collaboration, rather than a lack of skill or motivation.

In many Egyptian healthcare institutions, documentation systems remain predominantly paper-based, and quality assurance processes are inconsistently enforced. As a result, even after effective training, institutional support is essential to ensure sustained and standardized application of tools like the CAM-ICU. These findings are supported by studies such as Awan et al. [47], which showed significant improvements in CAM-ICU documentation accuracy following training, and Balas et al. [48], who linked structured implementation with better patient outcomes and recordkeeping. Blevins and DeGennaro [46] further noted that although 79% of nurses performed delirium screening correctly, common errors underscored the need for both skill development and procedural reinforcement.

#### Implications

This study demonstrates that structured, interactive, and context-specific educational training can significantly improve ICU nurses’ knowledge, perceived self-confidence, comfort levels, and application of delirium assessment tools. In the Egyptian context, where delirium screening is underutilized and formal training is limited, implementing such educational programs could bridge critical gaps in nursing practice. Hospital administrators and nursing educators should prioritize incorporating delirium assessment modules into ongoing professional development and undergraduate nursing curricula. Moreover, institutional support, such as integrating electronic documentation tools and establishing routine monitoring systems, is vital for sustaining consistent CAM-ICU application in ICU settings.

### Limitations

This study has several limitations that should be considered when interpreting the findings. First, the sample size and setting may limit the generalizability of the results. The study was conducted in an Egyptian ICU setting, and variations in institutional policies, resources, and patient populations may influence the applicability of the findings to other contexts.

Second, the reliance on self-reported measures of knowledge and self-confidence may introduce bias. Participants may have overestimated or underestimated their competencies, and future studies could benefit from incorporating objective assessments, such as performance-based evaluations or clinical outcome measures. Finally, the study did not assess long-term knowledge retention or sustained changes in clinical practice. Future research should include follow-up assessments to evaluate the durability of training effects over time and their impact on patient care outcomes.

## Conclusion

This study demonstrates that targeted educational training significantly improves ICU nurses’ knowledge, perceived self-confidence, and comfort levels, and documentation accuracy in managing delirium using the CAM-ICU tool. The findings indicate that a structured, interactive training program can effectively bridge knowledge gaps and enhance clinical practices. The high rate of accurate delirium assessment and documentation post-training emphasizes the practical success of the intervention in promoting procedural adherence and enhancing the quality of patient care.

Further research is needed to validate these findings across diverse ICU settings and over extended periods. Future studies should explore the long-term training effects, including knowledge retention, behavioral consistency, and impact on clinical outcomes such as delirium incidence, duration, ICU length of stay, and mortality. Additionally, examining how demographic factors such as education level and years of experience influence the effectiveness of educational interventions would offer valuable insights. Exploring the use of technology-enhanced training modalities, such as e-learning and virtual simulation, may also provide scalable and adaptable solutions for improving delirium care across varied clinical contexts. Overall, this study reinforces the importance of continuous, context-specific education in enhancing ICU nurses’ competencies and improving delirium screening, detection, and documentation in Egyptian ICU settings.

## Data Availability

Due to privacy/ethical restrictions, the data supporting the findings of this study are not publicly available but can be accessed upon reasonable request from the corresponding author.
